# Strengths and limitations of HFpEF probability scores in the era of precision medicine

**DOI:** 10.3389/fmed.2026.1799451

**Published:** 2026-04-07

**Authors:** Michał Wilk, Tomasz Kotwica, Robert Zymliński, Piotr Gajewski

**Affiliations:** 1 Student Scientific Organization, Institute of Heart Diseases, Wroclaw Medical University, Wroclaw, Poland; 2 Institute of Heart Diseases, Wroclaw Medical University, Wroclaw, Poland

**Keywords:** atrial fibrillation, H2FPEF, HFA-PEFF, HFpEF, obesity, precision medicine, probability scores

## Abstract

**Background:**

Heart failure with preserved ejection fraction is a heterogeneous syndrome influenced by comorbidities, complicating diagnosis and treatment. Probability-based scales, such as HFA-PEFF and H2FPEF, are commonly used but have limitations in specific phenotypes, including atrial fibrillation and obesity.

**Methods:**

A structured literature review was conducted using PubMed, Scopus, Wiley Online Library, and Google Scholar. Keywords included “HFpEF,” “HFA-PEFF,” “H2FPEF,” “atrial fibrillation,” “obesity,” “probability scores,” and “precision medicine.” Relevant studies were screened to summarize the performance, strengths, and limitations of HFpEF probability scales and their implications for precision medicine.

**Results:**

HFA-PEFF and H2FPEF effectively capture typical HFpEF presentations and can rule out disease with low scores. Limitations arise in phenotypes with AF, which alters natriuretic peptide levels and echocardiographic parameters, and obesity, which impairs NP-based diagnostics. Integration of advanced phenotyping, including left atrial function and BMI-adjusted thresholds, may improve detection. Precision medicine strategies, such as GLP-1 receptor agonists for obesity-related HFpEF or AF ablation, highlight phenotype-specific therapeutic potential.

**Conclusion:**

HFpEF probability scores are valuable tools but must be interpreted in the context of individual phenotypes. Precision medicine approaches, combining phenotyping, comorbidity assessment, and targeted therapies, offer improved diagnostic and therapeutic strategies for this heterogeneous population.

## 1 Introduction

Heart failure with preserved ejection fraction (HFpEF) is a phenotype of heart failure (HF) that has recently undergone a revolution in our understanding of HFpEF as a disease (1–4). Based on numerous observations, the view is emerging that HFpEF is a heterogeneous clinical syndrome rather than a specific disease entity (1, 4–6). There is increasing recognition that the development and progression of HFpEF are strongly influenced by comorbidities, which seem to be the most important risk factors (1, 2, 5, 7–15). These include cardiovascular disease, metabolic disorders, pulmonary disease, renal disease, and other geriatric factors, including frailty (1, 10, 12, 16–23). This is one reason why the number of HFpEF diagnoses is increasing; more specifically, an aging population has a significantly higher risk of developing comorbidities and is more exposed to disease progression and possible decompensation, which significantly worsens the prognosis and outcomes (20, 24). Moreover, the diagnostic tools currently available for diagnosing HFpEF are continually being refined to increase detection rates, which is another reason for the significant increase in this phenotype in the HF population (14, 25–28). An additional important factor in diagnosing HFpEF is left ventricular diastolic dysfunction (1, 11, 29–32). In light of recent observations, it is important to note that this dysfunction is not a universal marker for all individuals with HFpEF (1, 11). At the same time, it may result from several overlapping mechanisms stemming from comorbidities rather than from HF itself (1, 32). For instance, atrial fibrillation (AF) appears to play a very important role in atrioventricular filling disorders, but it is difficult to definitively determine in each case whether AF is an initial problem in a patient with HFpEF or rather a consequence of the progression of this HF phenotype, especially if HFpEF remains undiagnosed (1, 22, 29, 32). Another issue is that, according to the current guidelines (published as the 2023 Focused Update of the 2021 ESC Guidelines for the diagnosis and treatment of acute and chronic heart failure), the only disease-modifying therapy for patients with HFpEF is sodium-glucose transporter type 2 inhibitors (SGLT-2i) (3). Therefore, therapeutic strategies are largely based on mitigating known risk factors for HFpEF, including treating comorbidities such as AF, obesity, hypertension, and type 2 diabetes mellitus (T2DM) (1–4). A direct consequence of this approach is a therapeutic direction based on phenotyping patients with HFpEF into different subtypes that may benefit from carefully selected medical interventions aimed at improving each patient’s overall condition (1, 5, 12, 20). This model of individualized therapy based on specific phenotypes within HFpEF appears to be legitimated by the best treatment outcomes to date, further supporting the notion that each patient with HFpEF develops for a slightly different reason and therefore needs to receive individualized therapy to achieve the greatest benefit (1, 5, 18–20). In parallel with advances in phenotyping, HF research is increasingly shifting toward a precision medicine framework, integrating clinical characteristics, imaging, biomarkers, functional testing, and molecular profiling to tailor diagnosis and therapy. In HFpEF, this paradigm is particularly relevant given the heterogeneity of underlying pathophysiological mechanisms and treatment responses. However, currently available diagnostic algorithms were largely developed before the widespread implementation of precision medicine strategies and may insufficiently capture this complexity.

This diagnostic complexity has prompted the development of probability-based algorithms, such as HFA-PEFF and H2FPEF (33, 34). However, despite their widespread adoption, it is increasingly recognized that commonly used HFpEF probability scales have important limitations, particularly with respect to their performance across distinct clinical phenotypes and their alignment with emerging precision medicine strategies (12, 28, 29). This is due to the fact that the phenogroups differ significantly in terms of clinical profile, biomarkers, cardiac and vascular remodeling, which secondarily influences the differences in prognosis and response to treatment (1, 12, 29). Therefore, the factors taken into account in the above-mentioned scales may be insufficient, especially in the context of overlapping symptoms, thus, HFpEF prevalence may be underestimated when probability scales are applied in isolation, particularly in patients with atypical clinical presentations, overlapping comorbidities, or exercise-induced hemodynamic abnormalities (28, 29, 35). In this article, we present specific cases of phenotypes in which HFpEF should be considered in a broader perspective than just diagnosing it using available algorithms, and we also present the strengths and weaknesses of diagnostic tools such as probability scales. We also critically evaluate the strengths and limitations of current HFpEF probability scores in the context of emerging precision medicine approaches. We focus on phenotype-specific diagnostic challenges, discuss clinical scenarios in which standard algorithms may be insufficient, and highlight opportunities for more individualized diagnostic and therapeutic strategies.

## 2 Methods

We conducted a narrative review using PubMed, Scopus, Wiley Online Library, and Google Scholar. Search terms included “HFpEF,” “heart failure with preserved ejection fraction,” “HFA-PEFF,” “H2FPEF,” “atrial fibrillation,” “obesity,” “probability scale,” and “precision medicine.” Inclusion criteria focused on studies examining the diagnostic performance of HFpEF probability scales, phenotype-specific limitations, and the application of a precision medicine approach. Articles were screened for relevance, favoring recent publications, guideline updates, and studies addressing hemodynamic, echocardiographic, and biomarker-based assessments. The selection of studies was performed by thematic relevance, with particular emphasis on recent high-quality publications, guideline updates, and clinically meaningful data relevant to HFpEF probability assessment and precision medicine strategies.

## 3 HFpEF probability scores: what they capture well

The H2FPEF and HFA-PEFF probabilistic scales were developed in response to the heterogeneity and diagnostic difficulty of the HFpEF patient subpopulation (1, 33, 34). Their aim is to assess the probability of developing HFpEF in patients with dyspnea, using parameters such as comorbidities occurrence, echocardiographic and hemodynamic parameters, and, in the case of the HFA-PEFF scale, cardiac biomarkers such as natriuretic peptides (NPs) [NT-pro-BNP or BNP] (33, 34). These scales are largely based on the “typical” presentation of HFpEF, which is characterized by features such as older age, obesity, arterial hypertension, AF, elevated filling pressure, and left atrial remodeling (33, 34). In clinical practice, these tools have many applications, such as ruling out the disease with a low score or identifying uncertain patients requiring further diagnostics, including stress or invasive testing (26). Therefore, this section focuses on the key clinical strengths and practical advantages of the described scales.

For patients reporting dyspnea on exertion, both scales appear to capture the key mechanisms of dyspnea in HFpEF well (36). Higher scores on both scales were associated with impaired LV systolic and diastolic function, increased estimated LV filling pressures, reduced cardiac output, and concomitant right ventricular systolic dysfunction during peak exercise (36). This indicates that, despite being based on resting parameters, both scores indirectly reflect exercise-induced hemodynamic impairment (36). Additionally, the H2FPEF scale was better at predicting actual exercise intolerance, measured by both exercise duration and peak oxygen consumption (VO₂), achieving moderate but significant discriminatory ability in identifying reduced aerobic capacity. Summarizing, the H2FPEF score predicted reduced aerobic capacity (AUC 0.71, *p* = 0.0005), whereas the HFA-PEFF score did not (*p* = 0.07) (36). These findings suggest that HFA-PEFF better describes the structural-biochemical features of HFpEF than its functional consequences (36). An important aspect of the cited analysis is also the very high negative predictive value of both scales at low scores (0–1 point), approximately 94% for H2FPEF and 100% for HFA-PEFF, respectively (36). These results can be interpreted as meaning that a very low score on the scale has a high power in ruling out HFpEF and can be used to limit further costly procedures, as the probability of confirming the diagnosis of HFpEF remains extremely low (36).

To clearly indicate in which clinical situations which scale works best, one should take a look at the analysis prepared by Yoon et al. (29). Among the 992 patients included in the analysis, the percentage of confirmed HFpEF ranged from 28.5% according to the 2021 ESC guidelines, to 21.1% according to the HFA-PEFF scale, to 4.7% according to the H2FPEF scale. Comparing HFA-PEFF and H2FPEF scores, 40.2% of patients were classified into different HFpEF probability categories, and the same diagnosis according to the three algorithms was limited to 3.5% (29). This shows significant discrepancies between the use of specific scales, which requires extraordinary attention and discernment on the part of the clinician as to when a particular scale may prove most useful. Stating that the usefulness of both scales differs significantly and covers different scope of clinical cases, specific observations should be cited—the HFA-PEFF scale is characterized by high sensitivity in excluding HFpEF (pooled SEN 0.96) and good specificity in the confirmatory approach (pooled SPE 0.86) (37). What is more, higher scores correlate with an increased risk of death from all causes (HR 1.39), (*p* < 0.001) (37). While the H2FPEF scale, thanks to the simple combination of clinical data and two basic echocardiographic parameters, works well in everyday practice as a “gate-keeping” tool in simpler cases. HFA-PEFF offers a more nuanced, multidimensional assessment that incorporates biomarkers, morphology, and cardiac function, improving diagnostic accuracy in cases suspected of having HFpEF (29, 37). Overall, both scores provide valuable support to clinicians, enabling a more systematic approach to diagnosing HFpEF- HFA-PEFF is particularly useful in patients with a moderate probability of HFpEF as it provides a more detailed, multidimensional assessment, whereas H2FPEF is excellent for quickly ruling out HFpEF in simpler or low-risk cases (29, 37). Despite these important strengths, the performance of both scores may be substantially reduced in specific clinical scenarios and patient phenotypes, as discussed below.

## 4 Atrial fibrillation and HFpEF probability scores: diagnostic overlap and potential misclassification

Atrial fibrillation (AF) is one of the most prevalent arrhythmias in patients with HF, particularly in HFpEF, with reported prevalence ranging from 25 to 39% and increasing with the severity of diastolic dysfunction (38). Owing to its strong clinical association with HFpEF, AF has been incorporated into both the HFA-PEFF and H2FPEF scoring systems. However, its presence substantially complicates the diagnostic evaluation of HFpEF, as symptoms, biomarker elevations, and echocardiographic abnormalities may result from AF itself rather than from underlying ventricular dysfunction (38). Consequently, AF may be considered a “masking phenotype” that systematically influences the performance of probability-based diagnostic algorithms. AF independently increases natriuretic peptide concentrations, interferes with echocardiographic assessment of diastolic function due to the absence of atrial contraction and cycle-length variability, and produces symptoms that closely overlap with those of HFpEF, including exertional dyspnea and exercise intolerance (39–43). However, the magnitude and consistency of this effect across broader and more heterogeneous HFpEF populations have not been fully established. As a result, the reliability of both HFA-PEFF and H2FPEF scores in patients with AF remains controversial. In this context, Ariyaratnam et al. investigated the diagnostic performance of these scores in patients with coexisting HFpEF and AF using invasive hemodynamic assessment as the reference standard (40). HFpEF was confirmed in 73.3% of patients based on mean left atrial pressure measured at rest and after volume loading, indicating that a substantial proportion of patients with AF harbor subclinical or underdiagnosed HFpEF that may not be detected by noninvasive methods. In this study, a high HFA-PEFF score (≥5 points) yielded a sensitivity of 40% and a specificity of 91%, whereas a high H2FPEF score (≥6 points) achieved a sensitivity of 69% and a specificity of 66% (40). Receiver operating characteristic analysis demonstrated no significant difference between the two algorithms (HFA-PEFF AUC = 0.663 vs. H2FPEF AUC = 0.707; *p* = 0.636), indicating comparable overall discriminatory performance relative to the invasive gold standard. It should be noted that reported diagnostic performance metrics originate from studies using different reference standards, including invasive hemodynamic assessment, guideline based criteria, or clinically confirmed diagnosis. Therefore, direct comparison of sensitivity, specificity, or AUC across studies should be interpreted with caution. Notably, despite the high prevalence of invasively confirmed HFpEF, only 31.7 and 60% of patients were classified as having a high probability of HFpEF by HFA-PEFF and H2FPEF, respectively (40). These findings underscore the superior diagnostic accuracy of invasive left atrial pressure assessment in this population and highlight the vulnerability of algorithm-based approaches to AF-related confounding (44). Differences in reference standards may partly explain variability in reported performance, highlighting the need to contextualize findings within the methodological framework of each study.

Poor agreement between HFA-PEFF and H2FPEF has also been reported in broader patient populations, with concordance varying according to clinical characteristics such as AF status, body mass index, and natriuretic peptide levels (38, 39). Murat et al. demonstrated that discordance between the two scores is particularly pronounced in AF-dominant phenotypes, reflecting fundamental differences in their underlying pathophysiological assumptions (12). In the HFA-PEFF algorithm, AF substantially increases the score through both biochemical components, driven by elevated natriuretic peptide levels, and structural components, particularly left atrial enlargement. This may lead to an upward shift of patients into higher probability categories regardless of true diastolic dysfunction severity, thereby reducing phenotypic discrimination (12). In contrast, the H2FPEF score incorporates AF in a more selective and indirect manner, placing greater emphasis on age, obesity, and hypertension. Consequently, patients with AF may receive lower probability estimates when evaluated using H2FPEF compared with HFA-PEFF, despite similar clinical profiles (12). This low concordance has important clinical implications, as it may result in overdiagnosis of HFpEF when using HFA-PEFF and underdiagnosis when applying H2FPEF. Nevertheless, these potential patterns of misclassification are primarily inferred from selected cohorts, and their generalizability to unselected real-world populations remains uncertain. Collectively, these observations indicate that AF is not merely a comorbidity but a dominant phenotype modifier that systematically alters the diagnostic performance of probability-based tools, necessitating cautious interpretation and phenotype-specific diagnostic strategies (12).

Consistent with these findings, available evidence indicates limited agreement between both scores and invasively assessed filling pressures in patients with AF (12, 40). Even modest AF-related distortion of key algorithm components may translate into substantial diagnostic discrepancies. One potential strategy to improve diagnostic performance in this setting involves integrating parameters that directly reflect left atrial function, such as indexed minimal left atrial volume (LAVi_min) (42). Bonelli et al. demonstrated that incorporation of LAVi_min significantly improved discrimination of elevated filling pressures, increasing the C-statistic from 0.51 to 0.70 for H2FPEF and from 0.53 to 0.70 for HFA-PEFF (42). Such refinements may enhance diagnostic accuracy in AF-dominant phenotypes and mitigate misclassification related to arrhythmia-related confounding. Importantly, prospective validation studies specifically designed to assess the performance of HFpEF probability scores in AF-dominant phenotypes are limited, and further large-scale investigations are required to clarify the true clinical impact diagnostic distortion.

## 5 Obesity-associated HFpEF: limitations of natriuretic peptide–based diagnostic scores

Obesity represents one of the most prevalent and clinically relevant comorbidities in HFpEF and constitutes one of its dominant phenotypes (7, 9, 15, 18, 19, 21, 45–47). Beyond increased body mass index, this phenotype is characterized by distinct metabolic, hormonal, inflammatory, and hemodynamic alterations that differentiate it from other forms of HFpEF (1, 12, 18, 19, 48). Excess adipose tissue secretes a broad range of proinflammatory cytokines, promoting chronic low-grade systemic inflammation (“smoldering inflammation”), which contributes to myocardial fibrosis, endothelial dysfunction, and neurohormonal activation driven by sympathetic overactivity (48, 49). In parallel, obesity is strongly associated with insulin resistance, microvascular dysfunction, and cardiometabolic comorbidities, including hypertension, coronary artery disease, chronic kidney disease, and T2DM (7, 9, 18, 19, 23, 34, 45, 46). Collectively, these mechanisms not only increase the risk of HFpEF development but also modify disease progression, frequently leading to more severe exercise intolerance and poorer therapeutic responses (1, 9, 18). From a diagnostic perspective, patients with obesity and HFpEF exhibit significantly lower circulating concentrations of BNP and NT-proBNP despite comparable hemodynamic burden relative to individuals with normal body mass index (50). This phenomenon is largely attributed to increased expression of natriuretic peptide clearance receptor-C (NPR-C) in adipose tissue, which enhances peptide removal from the circulation (51) processing, resulting in diminished release of biologically active peptides. Structural factors, including increased epicardial and pericardial fat, may further attenuate perceived left ventricular wall stress, thereby reducing the stimulus for natriuretic peptide secretion (51). Together, these mechanisms underlie the so-called “natriuretic handicap,” characterized by impaired natriuretic peptide signaling and diminished biomarker responsiveness in obese individuals (52).

Clinical studies indicate that this biomarker attenuation has important diagnostic implications. Chen et al. reported that approximately 13.7% of hospitalized patients with HFpEF present with NT-proBNP levels below 300 ng/L, a subgroup predominantly composed of younger, often obese men (53). Similar observations have been reported in other cohorts (40, 53). Importantly, patients with obesity may be preferentially classified into low or intermediate probability categories by both HFA-PEFF and H2FPEF algorithms despite invasively confirmed HFpEF, even in the presence of concomitant AF, which would be expected to elevate natriuretic peptide levels (40). These findings suggest that obesity acts as a strong phenotype modifier, substantially reducing the diagnostic sensitivity of natriuretic peptide–based scoring systems and promoting underestimation of HFpEF probability. However, the extent to which this phenomenon translates into systematic underdiagnosis across diverse clinical settings remains incompletely defined. To date, no large prospective studies have systematically evaluated the performance of HFA-PEFF and H2FPEF scores across different body mass index strata, and current evidence remains largely indirect or hypothesis-generating. Given the rapidly increasing prevalence of obesity among patients with HFpEF, there is growing interest in the development of BMI-adjusted diagnostic thresholds and phenotype-specific cut-off values for natriuretic peptides (54). Based on available evidence, we propose that recalibration of natriuretic peptide–dependent components, particularly within the HFA-PEFF algorithm, may represent a pragmatic strategy to improve diagnostic accuracy in obese patients (33). At present, however, this concept remains hypothesis generating and has not been prospectively validated in large, phenotype-stratified cohorts. However, incorporation of complementary parameters like oncostatin M, seems to improve the diagnostic accuracy of the HFA-PEFF scale as well (55). Analogous to the use of rhythm-specific cut-offs in atrial fibrillation, BMI-adapted thresholds could enhance sensitivity without compromising specificity; however, this concept requires prospective validation. Therefore, BMI adjusted natriuretic peptide thresholds should currently be regarded as a proposed research direction rather than an established clinical standard. Overall, while the pathophysiological rationale supporting obesity-related attenuation of natriuretic peptide based algorithms is compelling, current evidence is largely derived from observational and subgroup analyses, underscoring the need for prospective phenotype specific validation.

## 6 Discussion and implications for precision medicine

Heterogeneity of HFpEF phenotypes combined with limitations resulting from the use of probabilistic scales in the diagnosis of HFpEF shows the need for a comprehensive approach to each case in accordance with the assumptions of so-called precision medicine (1, 20). Traditional diagnostic algorithms, although they may often seem useful in everyday clinical practice, carry hidden traps that should be remembered when using the described tools. More specifically, it should be remembered that the H2FPEF and HFA-PEFF scales have a limited ability to recognize specific phenotypes, such as those dominated by AF or obesity, in accordance with reality, and therefore the results obtained with their use may be either overestimated or underestimated (39, 40, 51, 52, 54, 56). AF has been shown to systematically modify HFA-PEFF and H2FPEF scores by increasing NPs levels and distorting echocardiographic parameters (39, 40), while obesity leads to a so-called “natriuretic handicap,” which reduces the reliability of NPs-based indices and complicates risk stratification in this subgroup (51, 52). These examples highlight the importance of individualized diagnostics that consider the impact of comorbidities, specific pathophysiological mechanisms, and the patient’s biomarker profile, rather than relying solely on generalized scales. For example, in AF dominant phenotypes, clinicians may consider early functional assessment with diastolic stress echocardiography or invasive hemodynamic testing when noninvasive probability scores appear incosistnet with symptom burden. Additionaly, in obesity dominant phenotypes with unexpectedly low natriuretic peptide levels, diagnostic interpretation should account for potential biomarker attenuation before excluding HFpEF. It is important to remember that determining the underlying cause: HF or a comorbidity associated with HF can often be difficult, if not impossible. However, this should not compromise the thoroughness of the diagnostic process, especially in questionable cases (20). In practical terms, this implies that probability scores should serve as an initial screening tool rather than a definitive diagnostic endpoint. In patients with intermediate scores or discordance between clinical presentation and calculated probability, further phenotype oriented evaluation should be considered before establishing or excluding the diagnosis of HFpEF.

Precision medicine enables the integration of patient data from many areas, namely clinical parameters, cardiac imaging parameters, hemodynamics, genetics and molecular profiles, making it possible to create personalized diagnostic and therapeutic paths. For instance, the described modification related to the addition of LA function such as LAVi min may have the benefit of improving the detection of HFpEF in patients with AF, demonstrating how advanced phenotyping can increase the accuracy of diagnosis (42). Similarly, recognizing obesity as a distinct phenotype, with specific metabolic, inflammatory, and hemodynamic changes, may lead to the development of BMI-adjusted diagnostic scales or to the use of specific cut-off points, as we suggested for NPs, improving the detection of HFpEF in this patient population (54). Conceptually, a precision guided diagnostic pathway may follow a model based on following steps: 1. initial probability assessment using HFA-PEFF or H2FPEF; 2. identification of dominant modifying phenotypes (e.g., AF, obesity, metabolic syndrome); 3. reassessment of the reliability of score components within that phenotype context; and 4. selective escalation to advanced imaging or invasive hemodynamic confirmation in cases of diagnostic uncertainty.

When it comes to the therapeutic application of precision medicine, it is worthwhile to consider therapies targeting specific phenotypes. Although SGLT2i are currently the only disease-modifying therapy with proven efficacy across the entire spectrum of HFpEF (3), other interventions may hold promise in selected subgroups. In obesity-related HFpEF, GLP-1 receptor agonists which reduce body weight, increase insulin sensitivity, and improve cardiovascular function may represent a phenotype-specific therapy (7, 19, 20). Similarly, in patients with AF, ablation can improve atrioventricular function and HFpEF symptoms (8, 32). This approach demonstrates the potential of precision medicine, where treatment is tailored to the phenotype and comorbidities, not just ejection fraction. In this framework, phenotype identification not only refines diagnosis but may also influence therapeutic prioritization, guiding clinicians toward rhythm control strategies in AF dominant HFpEF or weight reduction oriented interventions in obesity associated disease.

A recently proposed diagnostic model, the HFpEF-ABA score, incorporate age, BMI, and history of AF, aiming to improve the detection of HFpEF in phenotypes dominated by obesity and atrial dysfunction (57). Although still under investigation, the HFpEF-ABA score represents a step toward precision medicine in HFpEF and highlights the potential utility of phenotype adjusted diagnostic algorithms. Its inclusion underscores the need for future studies to validate and integrate such approaches into clinical practice (57).

In summary, integrating detailed phenotyping, comorbidity assessment, and individualized therapeutic strategies is essential to improving the diagnosis and treatment of HFpEF. Precision medicine offers the opportunity to go beyond a one-size-fits-all approach, tailoring interventions to the pathophysiology and specific patient characteristics, ultimately improving outcomes in this heterogeneous population.

## References

[1] Anker SD Usman MS Anker MS Butler J Böhm M Abraham WT . Patient phenotype profiling in heart failure with preserved ejection fraction to guide therapeutic decision making. A scientific statement of the heart failure association, the European heart rhythm Association of the European Society of cardiology, and the European Society of Hypertension. Eur J Heart Fail. (2023) 25:936–55. doi: 10.1002/ejhf.2894, 37461163

[2] Gajewski P Zymlinski R Biegus J. The critical role of comorbidities in managing heart failure with preserved ejection fraction (HFpEF). ESC Heart Fail. (2025) 12:1541–3. doi: 10.1002/ehf2.15169, 39548848 PMC12055417

[3] McDonagh TA Metra M Adamo M Gardner RS Baumbach A Böhm M . 2023 focused update of the 2021 ESC guidelines for the diagnosis and treatment of acute and chronic heart failure. Eur Heart J. (2023) 44:3627–39. doi: 10.1093/eurheartj/ehad195, 37622666

[4] Beghini A Sammartino AM Papp Z Von Haehling S Biegus J Ponikowski P . 2024 update in heart failure. ESC Heart Fail. (2025) 12:8–42. doi: 10.1002/ehf2.14857, 38806171 PMC11769673

[5] Bonfioli GB Pagnesi M Calò L Metra M. Towards a phenotype profiling of the patients with heart failure and preserved ejection fraction. Eur Heart J Suppl. (2025) 27:i115–21. doi: 10.1093/eurheartjsupp/suae095 39980782 PMC11836726

[6] Cohen JB Schrauben SJ Zhao L Basso MD Cvijic ME Li Z . Clinical Phenogroups in heart failure with preserved ejection fraction. JACC Heart Fail. (2020) 8:172–84. doi: 10.1016/j.jchf.2019.09.009, 31926856 PMC7058514

[7] Cimino G Vaduganathan M Lombardi CM Pagnesi M Vizzardi E Tomasoni D . Obesity, heart failure with preserved ejection fraction, and the role of glucagon-like peptide-1 receptor agonists. ESC Heart Fail. (2024) 11:649–61. doi: 10.1002/ehf2.14560 38093506 PMC10966224

[8] Fauchier L Bisson A Bodin A. Heart failure with preserved ejection fraction and atrial fibrillation: recent advances and open questions. BMC Med. (2023) 21:54. doi: 10.1186/s12916-023-02764-3, 36782248 PMC9926737

[9] Hobbach AJ Brix TJ Weyer-Elberich V Varghese J Reinecke H Linke WA. Obesity and comorbidities in HFpEF: a retrospective cohort analysis in a university hospital setting. J Clin Med. (2025) 14:3348. doi: 10.3390/jcm14103348, 40429344 PMC12112532

[10] Huang W Cheng H Yu W Guo C Chiang C Chen C . The ventilatory abnormalities and prognostic values of H_2_ FPEF score in dyspnoeic patients with preserved left ventricle systolic function. ESC Heart Fail. (2020) 7:1872–9. doi: 10.1002/ehf2.12754, 32488965 PMC7373923

[11] Laenens D Zegkos T Kamperidis V Wong RCC Li TY Sia C . Heart failure risk assessment in patients with hypertrophic cardiomyopathy based on the H_2_ FPEF score. Eur J Heart Fail. (2024) 26:2173–82. doi: 10.1002/ejhf.3413 39189810

[12] Murat S Murat B Yalvac HE Durmaz FE Inan D Celik A . The value of the HFA-PEFF and H2FPEF scores in determining the phenotypes and comorbidity burden in heart failure with preserved ejection fraction. Clin Physiol Funct Imaging. (2025) 45:e70019. doi: 10.1111/cpf.70019, 40539494

[13] Seoudy H Von Eberstein M Frank J Thomann M Puehler T Lutter G . HFA-PEFF score: prognosis in patients with preserved ejection fraction after transcatheter aortic valve implantation. ESC Heart Fail. (2022) 9:1071–9. doi: 10.1002/ehf2.13774, 35092186 PMC8934930

[14] Uchmanowicz I Lisiak M Ponikowski P Jankowska EA Mysiak A Wleklik M. A two-phase approach to identifying HFpEF in heart failure patients: risk score evaluation and decision tree development. ESC Heart Fail. (2025) 12:3079–85. doi: 10.1002/ehf2.15333, 40433849 PMC12287802

[15] Van Essen BJ Dan NAE Tharsana GN Kaur P Emmens JE Ouwerkerk W . Obesity and inactivity cluster the strongest risk factor for the development of heart failure in a population-based study. Int J Cardiol. (2026) 442:133914. doi: 10.1016/j.ijcard.2025.133914, 40976358

[16] Rakisheva A Soloveva A Shchendrygina A Giverts I. Heart failure with preserved ejection fraction and frailty: from young to superaged coexisting HFpEF and frailty. Int J Heart Fail. (2024) 6:93–106. doi: 10.36628/ijhf.2023.0064, 39081641 PMC11284337

[17] Zainul O Marshall D Lau JD Kelly B Zarzuela K Damluji A . Comparison of physical frailty assessments in heart failure with preserved ejection fraction. JACC: Advances. (2024) 3:101395. doi: 10.1016/j.jacadv.2024.101395, 39736919 PMC11683403

[18] Borlaug BA Jensen MD Kitzman DW Lam CSP Obokata M Rider OJ. Obesity and heart failure with preserved ejection fraction: new insights and pathophysiological targets. Cardiovasc Res. (2023) 118:3434–50. doi: 10.1093/cvr/cvac120, 35880317 PMC10202444

[19] Zheng W Qi Q Li J He C Fan H. Heart failure with preserved ejection fraction and obesity: emerging metabolic therapeutic strategies. Diabetol Metab Syndr. (2025) 17:336. doi: 10.1186/s13098-025-01917-z, 40826119 PMC12359955

[20] Rasalam R Sindone A Deed G Audehm RG Atherton JJ. State of precision medicine for heart failure with preserved ejection fraction in a new therapeutic age. ESC Heart Fail. (2025) 12:1544–57. doi: 10.1002/ehf2.15205, 39844745 PMC12055434

[21] Redfield MM Borlaug BA. Heart failure with preserved ejection fraction: a review. JAMA. (2023) 329:827–38. doi: 10.1001/jama.2023.2020, 36917048

[22] Vasilev V Popovic D Ristic GG Arena R Radunovic G Ristic A. H_2_ FPEF score predicts atherosclerosis presence in patients with systemic connective tissue disease. Clin Cardiol. (2021) 44:946–54. doi: 10.1002/clc.23621, 34075600 PMC8259163

[23] Hamatani Y Iguchi M Kato T Inuzuka Y Tamaki Y Ozasa N . Importance of non-cardiovascular comorbidities in atrial fibrillation and heart failure with preserved ejection fraction. ESC Heart Fail. (2025) 12:389–400. doi: 10.1002/ehf2.15093, 39305136 PMC11769661

[24] Akao K Imamura T Tanaka S Onoda H Ushijima R Sobajima M . Prognostic impact of modified H2FPEF score in patients receiving trans-catheter aortic valve replacement. J Clin Med. (2023) 12:5396. doi: 10.3390/jcm12165396, 37629434 PMC10455783

[25] Weerts J Amin H Barandiarán Aizpurua A Gevaert AB Handoko ML Dauw J . Webtool to enhance the accuracy of diagnostic algorithms for HFpEF: a prospective cross-over study. ESC Heart Fail. (2023) 10:3493–503. doi: 10.1002/ehf2.14525, 37724334 PMC10682885

[26] Parcha V Malla G Kalra R Patel N Sanders-van Wijk S Pandey A . Diagnostic and prognostic implications of heart failure with preserved ejection fraction scoring systems. ESC Heart Fail. (2021) 8:2089–102. doi: 10.1002/ehf2.13288, 33709628 PMC8120372

[27] Mu G Wang W Liu C Xie J Zhang H Zhang X . Combination of SVI/S′ and diagnostic scores for heart failure with preserved ejection fraction. Clin Exp Pharmacol Physiol. (2023) 50:677–87. doi: 10.1111/1440-1681.13782, 37203426

[28] Nikorowitsch J Bei Der Kellen R Kirchhof P Magnussen C Jagodzinski A Schnabel RB . Applying the ESC 2016, H2 FPEF, and HFA-PEFF diagnostic algorithms for heart failure with preserved ejection fraction to the general population. ESC Heart Fail. (2021) 8:3603–12. doi: 10.1002/ehf2.13532, 34459154 PMC8497222

[29] Yoon M Oh J Lee CJ Kang SM. Comparison of the 2021 ESC guideline with the HFA-PEFF and H2FPEF scores in diagnosing heart failure with preserved ejection fraction. Sci Rep. (2025) 15:22256. doi: 10.1038/s41598-025-01537-7, 40595806 PMC12215542

[30] Yavuz YE Soylu A Gürbüz AS. The relationship of systemic and pulmonary arterial parameters with HFpEF scores (H_2_ FPEF, HFA-PEFF) and diastolic dysfunction parameters in heart failure patients with preserved ejection fraction. J Clin Ultrasound. (2024) 52:39–50. doi: 10.1002/jcu.23572, 37904579

[31] Tomasoni D Aimo A Merlo M Nardi M Adamo M Bellicini MG . Value of the HFA-PEFF and H_2_ FPEF scores in patients with heart failure and preserved ejection fraction caused by cardiac amyloidosis. Eur J Heart Fail. (2022) 24:2374–86. doi: 10.1002/ejhf.2616, 35855616 PMC10087855

[32] Parwani AS Kossmann B Schweiger V. Targeting atrial fibrillation in HFpEF: the emerging role of pulsed field ablation. Front Physiol [Internet]. (2025) 16:1621118. doi: 10.3389/fphys.2025.1621118 40809291 PMC12343524

[33] Egashira K Sueta D Komorita T Yamamoto E Usuku H Tokitsu T . HFA-PEFF scores: prognostic value in heart failure with preserved left ventricular ejection fraction. Korean J Intern Med. (2022) 37:96–108. doi: 10.3904/kjim.2021.272, 34929994 PMC8747922

[34] Reddy YNV Carter RE Obokata M Redfield MM Borlaug BA. A simple, evidence-based approach to help guide diagnosis of heart failure with preserved ejection fraction. Circulation. (2018) 138:861–70. doi: 10.1161/CIRCULATIONAHA.118.034646, 29792299 PMC6202181

[35] Gil-Millan P Gimeno-Orna J Rodriguez-Padial L Muniz J Barrios V Anguita M . HfpEF as the predominant and underrecognized heart failure phenotype in type 2 diabetes: evidence from the DIABET-IC study. Cardiovasc Diabetol. (2025) 24:419. doi: 10.1186/s12933-025-02995-z, 41185008 PMC12581349

[36] Amanai S Harada T Kagami K Yoshida K Kato T Wada N . The H2FPEF and HFA-PEFF algorithms for predicting exercise intolerance and abnormal hemodynamics in heart failure with preserved ejection fraction. Sci Rep. (2022) 12:13. doi: 10.1038/s41598-021-03974-6, 34996984 PMC8742061

[37] Li S Zhu X Zhang Y Li F Guo S. Validation of heart failure algorithm for diagnosing heart failure with preserved ejection fraction: a meta-analysis. ESC Heart Fail. (2023) 10:2225–35. doi: 10.1002/ehf2.14421, 37292053 PMC10375189

[38] Gopinathannair R Chen LY Chung MK Cornwell WK Furie KL Lakkireddy DR . Managing atrial fibrillation in patients with heart failure and reduced ejection fraction: a scientific statement from the American Heart Association. Circ Arrhythm Electrophysiol [Internet]. (2021). 14:e000078. doi: 10.1161/HAE.0000000000000078 34129347

[39] Ariyaratnam JP Elliott AD Mishima RS Gallagher C Lau DH Sanders P. Heart failure with preserved ejection fraction: an alternative paradigm to explain the clinical implications of atrial fibrillation. Heart Rhythm O2. (2021) 2:771–83. doi: 10.1016/j.hroo.2021.09.015, 34988529 PMC8710629

[40] Ariyaratnam JP Mishima RS Kadhim K Emami M Fitzgerald JL Thiyagarajah A . Utility and validity of the HFA-PEFF and H2FPEF scores in patients with symptomatic atrial fibrillation. JACC Heart Fail. (2024) 12:1015–25. doi: 10.1016/j.jchf.2024.01.015, 38520461

[41] Hirano R Ikemura N Nguyen DD Jones PG Kimura T Katsumata Y . Clinical utility of the H2FPEF score in patients with early atrial fibrillation. JACC Heart Fail. (2024) 12:1302–5. doi: 10.1016/j.jchf.2023.12.011, 38363273

[42] Bonelli A Degiovanni A Beretta D Cersosimo A Spinoni EG Bosco M . H2FPEF and HFA-PEFF scores performance and the additional value of cardiac structure and function in patients with atrial fibrillation. Int J Cardiol. (2024) 413:132385. doi: 10.1016/j.ijcard.2024.132385, 39032577

[43] Foulkes SJ Moura-Ferreira S Milani M Bekhuis Y Falter M Delpire B . Impact of atrial fibrillation on hemodynamics, oxygen consumption and its Fick determinants in patients with HFpEF. J Card Fail. (2025). doi: 10.1016/j.cardfail.2025.12.007 41478588

[44] Wattanachayakul P Kittipibul V Salah HM Yaku H Gustafsson F Baratto C . Invasive haemodynamic assessment in heart failure with preserved ejection fraction. ESC Heart Fail. (2025) 12:1558–70. doi: 10.1002/ehf2.15163 39520094 PMC12055371

[45] van Dalen BM Chin JF Motiram PA Hendrix A Emans ME Brugts JJ . Challenges in the diagnosis of heart failure with preserved ejection fraction in individuals with obesity. Cardiovasc Diabetol. (2025) 24:71. doi: 10.1186/s12933-025-02612-z, 39920805 PMC11806779

[46] Packer M. Do obesity and visceral adiposity promote heart failure with reduced ejection fraction? Eur Heart J. (2025) 47:12–21. doi: 10.1093/eurheartj/ehaf645 PMC12765561 40891153

[47] Hathorn B Haykowsky MJ Almandoz J Pandey A Sarma S Hearon CM . Insights into the role of obesity in heart failure with preserved ejection fraction: pathophysiology and management. Can J Cardiol. (2025) 41:1764–76. doi: 10.1016/j.cjca.2025.03.015, 40122162 PMC13160563

[48] Packer M. The Adipokine hypothesis of heart failure with a preserved ejection fraction. JACC. (2025) 86:1269–373. doi: 10.1016/j.jacc.2025.06.055, 40886173 PMC12766646

[49] Wilk MM Wilk J Florek K Zimoch WJ. Smoldering inflammation: the silent flame driving heart failure. Dent Med Probl. (2025) 62:963–76. doi: 10.17219/dmp/210102 41186161

[50] Meijers WC Hoekstra T Jaarsma T Van Veldhuisen DJ De Boer RA. Patients with heart failure with preserved ejection fraction and low levels of natriuretic peptides. Neth Heart J. (2016) 24:287–95. doi: 10.1007/s12471-016-0816-8 26940695 PMC4796061

[51] Savarese G Schiattarella GG Lindberg F Anker MS Bayes-Genis A Bäck M . Heart failure and obesity: translational approaches and therapeutic perspectives. A scientific statement of the heart failure association of the ESC. Eur J Heart Fail. (2025) 27:1273–93. doi: 10.1002/ejhf.3676, 40328668 PMC12370599

[52] Hollstein T Schlicht K Krause L Hagen S Rohmann N Schulte DM . Effect of various weight loss interventions on serum NT-proBNP concentration in severe obese subjects without clinical manifest heart failure. Sci Rep. (2021) 11:10096. doi: 10.1038/s41598-021-89426-7, 33980890 PMC8115663

[53] Chen YY Liang L Tian PC Feng JY Huang LY Huang BP . Clinical characteristics and outcomes of patients hospitalized with heart failure with preserved ejection fraction and low NT-proBNP levels. Medicine (Baltimore). (2023) 102:e36351. doi: 10.1097/MD.0000000000036351, 38013260 PMC10681576

[54] Vaishnav J Chasler JE Lee YJ Ndumele CE Hu J Schulman SP . Highest obesity category associated with largest decrease in N-terminal pro-B-type natriuretic peptide in patients hospitalized with heart failure with preserved ejection fraction. J Am Heart Assoc. (2020) 9:e015738. doi: 10.1161/JAHA.119.015738 32750299 PMC7792252

[55] Sari H Keskin O Alsancak Y. Diagnostic significance of H2FPEF and HFA-PEFF scores with oncostatin M levels in heart failure with preserved ejection fraction. Herz. (2026). doi: 10.1007/s00059-025-05362-6 41528452

[56] Ouwerkerk W Tromp J Jin X Jaufeerally F Yeo PSD Leong KTG . Heart failure with preserved ejection fraction diagnostic scores in an Asian population. Eur J Heart Fail. (2020) 22:1737–9. doi: 10.1002/ejhf.1851 32378225

[57] Borlaug BA Zile MR Kramer CM Litwin SE Ye W Ou Y . HFpEF-ABA score is a more useful enrichment tool than natriuretic peptides in heart failure with preserved ejection fraction. Eur Heart J. (2025) 46:4607–9. doi: 10.1093/eurheartj/ehaf663, 40886160 PMC12614996

