# Correction: Strengths and limitations of HFpEF probability scores in the era of precision medicine

**DOI:** 10.3389/fmed.2026.1904490

**Published:** 2026-06-24

**Authors:** Michał Wilk, Tomasz Kotwica, Robert Zymliński, Piotr Gajewski

**Affiliations:** 1 Student Scientific Organization, Institute of Heart Diseases, Wroclaw Medical University, Wroclaw, Poland; 2 Institute of Heart Diseases, Wroclaw Medical University, Wroclaw, Poland

**Keywords:** atrial fibrillation, H2FPEF, HFA-PEFF, HFpEF, obesity, precision medicine, probability scores

An incorrect **Funding** statement was provided. The correct **Funding** statement reads:

“The author(s) declared that financial support was received for this work and/or its publication. This research was financed by a grant from Wroclaw Medical University IDUB.A46B.24.002. The presented research results were funded by the Development Strategy of Wroclaw Medical University entitled “UMW in the Light of Scientific Excellence 2024–2026”.”

The original version of this article has been updated.

